# Unhealthy Diets, Unhealthy Futures: How Modern Eating Patterns Endanger Maternal and Offspring Health

**DOI:** 10.3390/nu18142320

**Published:** 2026-07-15

**Authors:** Anallely López-Yerena, Victoria Pinto, Beatrice Maria Stella, Ezgi Yaşar, Miquel Camafort, Mei Qi Vives-Giralt, Francesc Casanovas-Garriga, Ana Maria Ruiz-Leon, Ramon Estruch, Rosa Casas

**Affiliations:** 1 Department of Internal Medicine, Hospital Clínic, Institut d’Investigació Biomèdica August Pi i Sunyer, Universitat de Barcelona, 08036 Barcelona, Spain; victoriasabinap@ub.edu (V.P.); camafort@ub.edu (M.C.); frcasang7@alumnes.ub.edu (F.C.-G.); amruiz@recerca.clinic.cat (A.M.R.-L.); restruch@ub.edu (R.E.); 2 Centro de Investigación Biomédica en Red (CIBER) de Fisiopatologia de la Obesidad y la Nutrición (CIBEROBN), Instituto de Salud Carlos III, 28029 Madrid, Spain; 3 Institut de Recerca en Nutrició i Seguretat Alimentaria (INSA-UB), University of Barcelona, 08921 Barcelona, Spain; 4 Departamento de Nutrición y Dietética, Escuela de Ciencias de la Salud, Facultad de Medicina, Pontificia Universidad Católica, Santiago 7820436, Chile; 5 Department of Medical Sciences, University of Turin, Corso Achille Mario Dogliotti, 14, 10126 Torino, Italy; beatricemaria.stella@unito.it; 6 Department of Nutrition and Dietetics, Faculty of Health Sciences, Izmir Kâtip Çelebi University, 35620 İzmir, Turkey; ezgiyasar99@gmail.com; 7 Faculty of Pharmacy and Food Sciences, University of Barcelona, 08028 Barcelona, Spain; mvivesgi13@alumnes.ub.edu

**Keywords:** ultra-processed foods, gut microbiota, early-childhood, pregnancy, maternal nutrition

## Abstract

Ultra-processed foods (UPFs) are increasingly prevalent in global diets and have been consistently associated with adverse health outcomes. Their consumption during sensitive life stages, such as pregnancy and early childhood, raises significant public health concerns due to potential intergenerational effects. This narrative review critically examines the impact of UPF consumption during pregnancy and early life, with a focus on maternal and child health outcomes, including alterations in gut microbiota composition. Accumulating evidence indicates that UPF consumption is linked to increased risks of obesity, type 2 diabetes, cardiovascular disease, and all-cause mortality. During pregnancy, high UPF intake is associated with poorer diet quality, excessive gestational weight gain, increased inflammation, and unfavorable neonatal outcomes, including altered microbiota transmission and impaired neurodevelopment. In early childhood, UPFs were linked to microbiota dysbiosis, obesity, micronutrient deficiencies, and allergic conditions. Notably, maternal dietary pattern strongly influences the early and sustained incorporation of UPFs into children’s diets. Overall, UPF consumption during pregnancy and early childhood represents a modifiable risk factor with far-reaching health implications. A deeper understanding of the dietary–microbiome–health axis is essential for developing effective nutritional strategies to optimize maternal and child health outcomes and reduce long-term disease risks.

## 1. Introduction

A growing body of evidence links high consumption of processed foods, particularly ultra-processed foods (UPFs), with adverse health outcomes. Epidemiological studies consistently associate elevated UPF intake with an increased risk of obesity, type 2 diabetes, cardiovascular disease, certain cancers, and all-cause mortality [1,2,3,4]. UPFs are typically rich in added sugars, saturated fats, salt, and various additives (e.g., flavorings, emulsifiers, non-sugar sweeteners), while being poor in fiber and essential micronutrients. Their global consumption continues to rise, driven by economic development, globalization, and transformations in food systems [5].

Optimal nutrition during pregnancy and lactation is critical for maternal and child health; however, UPF consumption remains high among pregnant and lactating women [6,7]. Pregnancy is characterized by dynamic changes in the maternal microbiome, which may influence maternal health, fetal development, and long-term offspring outcomes [7,8,9]. These microbiome shifts are modulated by factors such as diet, stress, infection, and metabolic conditions, with diet-induced dysbiosis linked to inflammation and metabolic dysfunction, potentially exerting intergenerational effects [10,11,12].

Diet is a key determinant of gut microbiota composition and diversity [13]. The Western dietary pattern, characterized by high UPF intake, has been associated with microbial imbalances, including increased Bacteroides and reduced Bifidobacterium, as well as disruptions in gut homeostasis induced by food additives [14,15]. Such alterations may contribute to increased intestinal permeability, inflammation, and a higher risk of metabolic and immune-related disorders in offspring [10,16].

Early-life gut microbiota plays a fundamental role in immune, metabolic, and neurodevelopmental processes. Its composition is shaped by prenatal, perinatal, and postnatal factors, including maternal microbiota, delivery mode, gestational age, feeding practices, antibiotic exposure, and environment [17,18]. Despite widespread recognition of the importance of adequate nutrition in early childhood, UPF consumption during this critical window is alarmingly high. Reports indicate that up to 50% of total energy intake among Chilean children under 5 years old is derived from UPFs [19]. Breastfeeding promotes microbiota enriched in beneficial genera such as Bifidobacterium and Lactobacillus, whereas formula feeding is associated with higher levels of potentially pathogenic bacteria, including Clostridium and Escherichia coli [20,21]. Emerging evidence suggests that supplementation of infant formula with specific probiotics may partially modulate these effects [22].

Although previous reviews have addressed the impact of UPFs on maternal and child health [23,24,25], their specific effects on the maternal and infant microbiome remain insufficiently explored. Therefore, this narrative review aims to critically examine UPF consumption during pregnancy and early life, focusing on its potential to alter gut microbiota and its short- and long-term implications for offspring health. In addition, we explore how psychological and social factors can modulate UPF consumption.

## 2. Methodology

For the purposes of this narrative review, inclusion criteria were restricted to studies conducted in human subjects with no restrictions on publication date, published in English. Animal studies, in vitro studies, and articles without relevant outcomes on maternal or early-life exposure to UPFs were excluded. A comprehensive literature search was conducted in Scopus, PubMed, and Google Scholar up to January 2026 to identify clinical and observational studies assessing the impact of ultra-processed food consumption during pregnancy and early life stages.

The search strategy combined controlled and free-text terms using Boolean operators. Search strings included combinations of keywords related to exposure and population, such as: (“ultra-processed foods” OR UPF) AND (“maternal diet” OR pregnancy OR gestation) AND (“offspring” OR infant OR “early childhood” OR neonatal) AND (microbiota OR “metabolic health” OR “fetal programming”). Additional relevant terms included dietary patterns, breastfeeding, infant formula, and microbiota transmission.

Reference lists of selected articles were also screened to identify additional studies not captured in the database search. Study selection was performed based on title and abstract screening followed by full-text assessment. A total of 84 studies were included in the final synthesis. These comprised 71 original research articles, 5 systematic reviews and meta-analyses, and 8 narrative reviews. Study selection followed a two-stage process consisting of title and abstract screening followed by full-text assessment. The final evidence base was synthesized thematically according to developmental stage and biological outcomes.

To structure the synthesis of evidence, the included studies were organized into three interrelated thematic domains: (1) current trends in UPF consumption, (2) health implications of UPF intake, and (3) a biopsychosocial pathway model describing potential mechanisms underlying the effects of UPFs. These domains reflect a conceptual continuum from exposure patterns to health outcomes and mechanistic pathways. A total of 27 original studies, 1 systematic review and meta-analysis, and 1 narrative review informed the first theme; 35 original studies, 2 systematic reviews and meta-analyses, and 4 narrative reviews informed the second theme; and 1 systematic review and meta-analysis and 1 narrative review contributed to the third theme. Some studies were included in more than one thematic category due to their overlapping relevance.

## 3. Current Trends in Ultra-Processed Food Consumption

The term UPF emerged in the 1980s, initially referring to highly processed convenience foods and snacks that were energy dense, nutrient-poor, and often contained synthetic additives such as emulsifiers, colorants, artificial sweeteners, and flavor enhancers [26]. Their widespread consumption and potential health effects have made UPFs a central focus in nutrition research. The NOVA food classification system categorizes foods based on their level of processing, dividing them into four groups: unprocessed or minimally processed foods (NOVA 1), processed culinary ingredients (NOVA 2), processed foods (NOVA 3), and UPFs (NOVA 4) [27].

UPFs are formulations of ingredients, mostly for exclusive industrial use, created through multiple industrial techniques and processes (hence the term ‘ultra-processed’) [28]. These products typically have an obesogenic nutrient profile: energy-dense, high in added sugars, unhealthy fats, and salt, and low in essential nutrients, depending on formulation. Common UPFs include carbonated soft drinks; sweet, fatty or salty packaged snacks; confectionery; industrial bread, pastry, biscuits, and cakes; margarine; sweetened breakfast cereals; fruit-flavored yogurts; and ready-made meat, cheese, pasta and pizza dishes.

Globally, UPF consumption is rising rapidly, with substantial increases reported in both high-income and middle-income countries. There is no universal threshold to define low or high consumption of UPFs, as most studies classify intake relatively within each population, typically using tertiles or quartiles of consumption. For this reason, cut-off values vary across studies; however, as a general reference, low consumption is usually around <15–20% of total daily energy intake, moderate consumption between 20 and 40%, and high consumption above >40–50% [29,30,31]. Regions such as North America, Europe, and Latin America show the highest consumption levels, while Asia, the Middle East, and Africa are experiencing accelerated growth [5]. This global trend is driven by the industrialization of food systems, technological advancements and globalization [32]. Across Europe, the contribution of UPFs to total energy varies widely, ranging from 14% to 44%, lowest in Italy and Romania, and highest in the UK and Sweden [33]. Another study examining household availability of UPFs found that UK households had the highest availability at 50.4% [34]. Data from the UK’s National Diet and Nutrition Survey Rolling Programme (NDNS) showed that 56.8% of caloric intake came from UPFs [34]. In France, the NutriNet-Santé cohort reported that 35.9% of total energy intake in adults was derived from UPFs [35]. In Spain, one study found that more than 20% of the energy comes from ultra-processed products [36], and another observed a 10.8% increase in UPF consumption between 1991 and 2008 [37].

In the Americas, ultra-processed products are a major component of modern diets. In the US, UPFs provided 54% of adults’ total energy intake in 2015–2018 [38]. In Canada, 48% of calories consumed in 2004 originated from highly processed foods [39]. In Brazil, UPFs accounted for 21.5% of total energy intake [40]. In Mexico, approximately 30% of dietary energy is derived from UPFs [41], although another study reported a wide range from 4.5% to 64.2%, depending on population subgroup (age, socioeconomic status, and educational level [42].

### 3.1. UPF Consumption in Pregnancy

Nutritional intake plays a fundamental role during pregnancy; however, contemporary fast-paced lifestyles and limited time frequently lead to the adoption of less healthy eating patterns. Higher UPF consumption consistently correlates with poorer diet quality. Pregnant women in the highest quintile of UPF intake had significantly lower intakes of protein, fiber, and multiple micronutrients (iron, magnesium, potassium, copper, zinc, selenium, folate), while consuming more trans fat and sodium [43]. Additionally, women with high UPF intake also had lower intakes of vitamin C, beta-carotene, vitamin B6, and potassium [7]. In a study of 2984 Norwegian pregnant women, high UPF consumption correlated with lower concentrations of nutritional biomarkers, such as carotenoids and vitamin A, during mid-pregnancy [44]. Furthermore, greater UPF consumption has been associated with reduced intake of essential nutrients, including protein and zinc, both of which are of particular importance during pregnancy [45,46].

A sub-study (*n* = 2377) of the INMA cohort [6] found that highly processed food products accounted for an average of 17% of the total diet among Spanish pregnant women, with sugar-sweetened beverages being the most consumed type of UPF, representing 40% of all cases. In the IMPACT BCN trial, women in the highest tertile of change in UPF consumption from second to third trimester had over twice the odds of preeclampsia (OR 2.29, 95% CI 1.06–4.97) [47]. This suggests that increasing UPF intake as pregnancy progresses may be particularly harmful. In another study carried out in Israel with a cohort of 206 pregnant women, the proportion of total energy derived from UPFs ranged from 15.6% to 43.4% [7]. Contrary to expectations that women adopt healthier eating patterns during pregnancy, a Brazilian cohort study found that UPF consumption increased from 28.9% preconception to 33% during pregnancy [48]. Older and more educated women showed lower increases in UPF consumption during pregnancy, while higher consumption was observed among women who were physically inactive before pregnancy, smoked during pregnancy, were multiparous, or had low pregestational weight [48].

### 3.2. UPF Intake During Early Childhood

According to World Health Organization recommendations [49], optimal breastfeeding practices include exclusive breastfeeding for the first six months of life and continued breastfeeding up to two years of age or beyond. These recommendations aim to support optimal infant growth, development and health [49]. As infants grow, complementary feeding introduces new foods to meet increased nutritional requirements. However, the expanding availability of UPFs targeted at infants poses a major challenge. Examples of highly processed baby foods include infant formula, follow-on and toddler/growing-up milks, baby food pouches, packaged baby snacks, baby cereals, commercial fruit yogurts, and ready-made baby meals.

Beyond the well-established health benefits of breast milk, breastfeeding has been associated with healthier childhood feeding practices, including timely introduction of solid foods, greater dietary diversity, and lower consumption of unhealthy foods [50,51,52]. However, UPF consumption during early childhood has increased substantially over the past two decades in the U.S. and globally, with UPFs now comprising the majority of total energy intake among young children. This trend represents a fundamental shift in dietary patterns, with significant variations by age, race/ethnicity, and geographic region.

In this regard, one study reported that Brazilian children aged 6–12 months and 12–24 months consumed approximately 2393 and 4054 kJ/d, respectively, representing about one-third of their total dietary energy intake [53]. The same study found a positive correlation between the higher energy intake from processed foods and UPFs and greater daily energy intake from saturated fat, total sugar and sodium. In the UK Gemini twin cohort, UPFs contributed 46.9% (±14.7%) of total energy intake at 21 months of age, and the principal UPF subgroups consumed by toddlers included yogurts, higher-fiber breakfast cereals, and wholegrain breads [54]. Among U.S. infants aged 6–12 months surveyed between 2009 and 2018, 65.5% were started on solid foods early, and a dietary pattern characterized by UPFs (versus natural or minimally processed foods) was identified [55]. UPFs consumed by US youths (2–19 years) in 2017–2018 contained significantly higher percentages of calories from carbohydrates (55.2% vs. 43.4% for non-UPFs) and added sugars (19.3% vs. 3.4%), but lower levels of fiber (0.67 g/100 kcal vs. 0.87 g/100 kcal) and protein (10.5% vs. 20.6%) [56].

Additionally, in New Zealand children, UPFs contributed a mean of 45% (95% CI 44–47%) of total energy intake at 12 months of age [57]. Among Chinese toddlers aged 6–36 months, 86.8% consumed UPFs, with the highest percentage consuming pastries (63.5%), followed by solid or semi-solid dairy products (58.8%), and reconstituted meat products (56.4%) [58]. Commercial snack foods comprising toddler foods (ages 12 to <36 months) available in Australian supermarkets, with 63% classified as ultra-processed and 77% assessed as sweet rather than savory [59].

Multiple interrelated factors contribute to UPF consumption in early childhood, including maternal characteristics, feeding practices, socioeconomic status, food environment, and marketing influences [59,60,61,62].

- **Maternal dietary patterns:** Maternal diet is one of the strongest predictors of children’s UPF intake. In the INMA cohort, high maternal UPF consumption during pregnancy was associated with a 3.5-fold increased risk of high UPF consumption in children at age 8 [63]. Similarly, in 4-year-old Spanish children, higher maternal UPF intake during pregnancy was associated with nearly triple the risk of high child UPF consumption [60]. Younger maternal age at pregnancy was also associated with higher child UPF intake, while maternal age ≥30 years was protective [60,64].
- **Breastfeeding duration and parental feeding behaviors:** Absence of breastfeeding was associated with nearly 4-fold higher odds of being in the highest tertile of UPF consumption among Brazilian children aged 6–24 months [65]. Longer breastfeeding duration was associated with lower UPF consumption in the SENDO cohort [66]. Lower parental monitoring of food intake and lower “Healthy Eating Guidance” scores were independently associated with UPF-dominant dietary patterns in Brazilian children [67].
- **Socioeconomic status:** The relationship between socioeconomic status and UPF consumption shows some geographic variation. In European and Latin American cohorts, lower maternal education and lower social class were consistently associated with higher child UPF intake. In the UK Gemini cohort, maternal postgraduate education was associated with approximately 9–10% less energy from UPF compared to no educational qualifications, making maternal education the strongest socioeconomic predictor [62]. Low maternal social class doubled the risk of high UPF consumption at age 8 in the INMA cohort [63]. However, US NHANES data (1999–2018) showed no significant disparities by parental education or family income-to-poverty ratio, suggesting UPF consumption is pervasive across all socioeconomic strata in the US [56].
- **Food environment and marketing:** The food environment and aggressive marketing are critical upstream drivers. UPFs are ubiquitously marketed through mass media, digital platforms, and sponsorships, far more than other food categories [68]. Companies build brand loyalty through tactics such as promoting healthy product lines and using child-friendly characters, particularly for infant and toddler products designed to resemble infant formulas [68]. Screen time and television viewing serve as proxies for marketing exposure. Specifically, children watching >1.5 h/day of TV had 65% higher odds of high UPF consumption, and this association persisted through age 12 [60,63].

These factors operate at individual, household, and societal levels to shape dietary patterns established in the first years of life.

## 4. Health Implications of UPF Consumption

### 4.1. Pregnancy

UPF consumption during pregnancy is associated with increased risk of gestational diabetes mellitus and preeclampsia, impaired fetal growth, and reduced maternal diet quality. The evidence shows dose-dependent relationships for some outcomes, though findings vary across populations. However, most of the available evidence is observational in nature and therefore does not establish causality.

Regarding metabolic health, a longitudinal study of 45 pregnant women in the U.S. reported that a higher percentage of energy intake from UPFs (PEI-UPF) was associated with increased gestational weight gain and greater neonatal body fat [69]. Another study identified an association between PEI-UPF and maternal obesity [7]. Similarly, PEI-UPF has been linked not only to excessive gestational weight gain, but also to higher blood circulating concentration of C-reactive protein during pregnancy and increased postpartum weight retention [70]. A dose–response systematic review and meta-analysis of 54 observational studies found that maternal UPF consumption before or during pregnancy was associated with a higher risk of gestational diabetes and preeclampsia [71]. In addition, the ingestion of UPFs prior to conception has been associated with an elevated probability of gestational diabetes, particularly in women aged 30 years or older [72]. However, another study reported no significant association between UPF consumption and maternal glycemic control in women with gestational diabetes, although it was associated with neonatal hypoglycemia [73]. Overall, these findings suggest a consistent association between higher UPF intake and adverse maternal metabolic outcomes, although some inconsistencies remain across studies.

Periconceptional UPF consumption has also been linked to impaired embryonic growth [74]. In terms of mental health, UPF consumption during pregnancy has been associated with higher levels of anxiety, stress, depressive symptoms, and feelings of sadness [75].

#### Gut Microbiota

Pregnancy induces significant, site-specific microbiota changes across the gut, vaginal, and oral compartments, driven by hormonal, immunological, metabolic, and dietary shifts. These changes are physiologically adaptive, supporting maternal metabolism and fetal development, but when dysregulated, they are associated with complications such as gestational diabetes, preeclampsia, and preterm birth [76,77,78].

During pregnancy, gut microbiota undergoes some of the most extensively characterized compositional and functional changes across gestation. In the first trimester, the gut microbial profile closely resembles that of healthy non-pregnant individuals [79]. However, as pregnancy progresses, particularly during the second and third trimesters, a progressive remodeling occurs, characterized by reduced alpha diversity and increased beta diversity, reflecting greater interindividual variability. By the third trimester, the gut microbiota exhibits a composition like that observed in individuals with metabolic syndrome and has been shown to induce metabolic syndrome-like features when transplanted into germ-free mice [79]. At the phylum level, these changes include an increase in *Pseudomonadota* (formerly *Proteobacteria*) and *Actinomycetota* (formerly *Actinobacteria*), together with a decline in butyrate-producing bacteria and an increased Firmicutes/Bacteroidetes ratio from the first to the second trimester [76,79]. At the genus level, an expansion of *Bifidobacterium*, *Akkermansia*, *Blautia*, *Collinsella*, and *Rothia* has been reported, with progesterone identified as a causal factor promoting Bifidobacterium growth [78,79]. Collectively, these microbial shifts are thought to represent adaptive physiological changes that support [78,79].

High UPF consumption during pregnancy is associated with unfavorable changes in gut microbiota composition [80]. When maternal obesity or high UPF is present, this profile is exacerbated, intensifying dysbiosis: beneficial bacteria decrease while pro-inflammatory species increase, which is associated with metabolic alterations such as insulin resistance and a higher risk of complications, including gestational diabetes [81,82].

A 2026 systematic review and meta-analysis of 29 studies involving 3077 pregnant women found that high-fat and Westernized diets (characteristic of UPF patterns) were linked to reduced microbial diversity and increased proinflammatory taxa including *Collinsella* and members of the *Lachnospiraceae* family, while high-fiber diets were associated with beneficial taxa such as *Roseburia* spp. and *Bifidobacterium* spp [80]. Specific dietary components common in UPFs, including saturated fat and added sugars, are associated with distinct microbial enterotypes and altered functional gene representation in the maternal gastrointestinal microbiome during pregnancy [83]. UPF consumption during pregnancy represents approximately 17–23% of total dietary intake in studied populations, with these foods characterized by high levels of saturated and trans fats, added sugars, sodium, and food additives including emulsifiers, artificial sweeteners, colorants, and preservatives [6,43,84].

**Figure 1:**
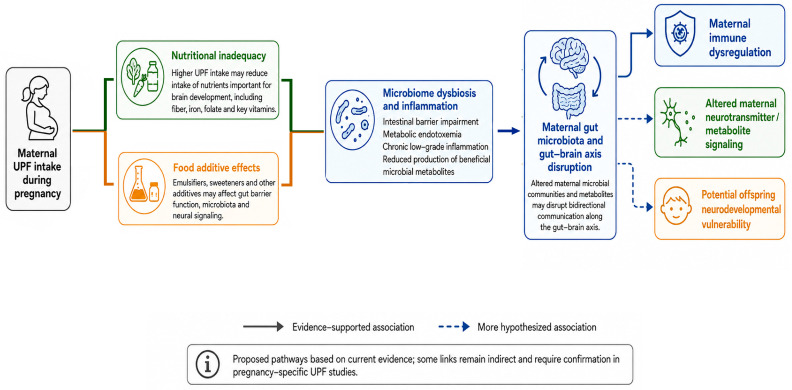
Proposed mechanism linking maternal UPF intake to gut–brain axis disruption.

These components directly alter gut microbiota functions and microbial metabolism, potentially impacting neural networks through the microbiota–gut–brain axis [84] as shown in Figure 1. Specifically, UPF consumption may disrupt the maternal–infant microbiome axis through several interconnected mechanisms, including intestinal barrier impairment, metabolic endotoxemia, chronic low-grade inflammation, and reduced production of beneficial microbial metabolites such as short-chain fatty acids (SCFAs). Food additives like emulsifiers, refined sugars, and artificial sweeteners can alter gut permeability, promote bacterial translocation, and activate inflammatory pathways. This may facilitate lipopolysaccharide (LPS) translocation into circulation, contributing to systemic inflammation and metabolic dysfunction during pregnancy. Moreover, reduced fiber intake associated with UPFs decreases SCFA production, impairing immune regulation and fetal programming, potentially increasing offspring susceptibility to metabolic and inflammatory disorders later in life [85,86].

### 4.2. Early Childhood

Evidence indicates that high UPF consumption during early childhood is associated with adverse health outcomes, including poor growth, increased risk of obesity, micronutrient deficiencies, and elevated blood pressure [87,88,89]. Additionally, high maternal UPF during breastfeeding does not appear to cancel breastfeeding’s core benefits outright, but it is associated with poorer breast milk nutritional quality, less favorable infant outcomes, and weaker protection against healthy diet formation [90]. In this sense, the strongest direct lactation evidence is still limited and mostly observational, so the main conclusion is attenuation rather than elimination of breastfeeding’s benefits [25,90].

As expected, inadequate nutrition is closely associated with obesity, a relationship partly mediated by the composition of the gut microbiome. Dietary patterns rich in unhealthy fats and refined sugars, characterized by the Western diet, have been associated with a specific microbial profile often described as an “obesogenic microbiota”, which possesses an increased capacity to extract energy from food, potentially contributing to weight gain [91]. The Growing Up in Singapore Towards Healthy Outcomes (GUSTO) cohort, which included 555 children aged 18 months, found that sugar-sweetened beverages were associated with a higher risk of adiposity and overweight/obesity [92]. Other studies have similarly demonstrated that UPF consumption is associated with a higher BMI and an adverse growth outcome in early childhood [93]. Moreover, a population-based cross-sectional study in Brazilian children aged 12–59 months reported that early introduction of UPFs was associated with overweight and anemia [94].

In children from low-income communities, increasing UPF consumption over time has been associated with less favorable lipid profiles [95]. Additionally, a positive association has been observed between UPF consumption and chronic subclinical inflammation, as measured by plasma CRP concentration among infants, regardless of excess weight [96]. Consumption of commercial baby food has also been linked to childhood food allergies [97], while dietary exposure to fructose, carbonated soft drinks, and high sugar intake has been associated with elevated risks of asthma, allergic rhinitis, and food allergies [97].

Additionally, one study reported that high UPF consumption in the third trimester was adversely associated with children’s verbal functioning at 4–5 years of age, a key cognitive domain related to verbal expression and conceptualization [6]. Moreover, a maternal diet rich in UPFs, saturated fats, and sugars has been shown to negatively influence offspring cognitive development, potentially contributing to the emergence of neurodevelopmental disorders [98]. Given these concerns, strategies have emerged to reduce free sugar, added sugars and UPFs during the first year of life. A multicenter randomized controlled trial involving 516 mother–child pairs in three state capitals of Brazil evidenced that dietary counseling effectively reduced early consumption of UPF and added sugar during infancy [99].

#### 4.2.1. Gut Microbiota

The first years of life constitute a critical window for gut microbiota establishment, during which microbial communities undergo rapid development and are highly sensitive to environmental influences. As this ecosystem matures, its plasticity progressively decreases, making early dietary exposures especially influential in shaping long-term microbial composition and, consequently, future health outcomes [100]. UPF consumption during this period may disrupt normal microbiota maturation, potentially increasing risk for metabolic diseases, immune dysfunction, and neurodevelopmental disorders [15,85].

A 2025 prospective birth cohort study of 728 infants from Brazil provides the most comprehensive evidence on UPF effects during the first year of life. Weaned children who consumed UPF showed significantly higher alpha diversity across all parameters (observed ASVs: *p* = 0.005; Shannon index: *p* = 0.036; Chao index: *p* = 0.026; Simpson index: *p* = 0.012) compared to breastfed children who did not consume UPF [101]. Most critically, breastfed children who did not consume UPF had significantly higher relative abundance of Bifidobacterium compared to weaned children who consumed UPF, while UPF-consuming children showed higher abundance of *Firmicutes*, *Blautia*, *Sellimonas*, and *Finegoldia* [101]. In Australian children aged 1–2 years, differential effects on specific Firmicutes-affiliated lineages were observed in response to frequent intake of either processed or unprocessed foods, highlighting that food processing itself, independent of specific nutrients, affects microbiota composition [102].

In this sense, Bifidobacterium species are essential for infant gut health, performing saccharolytic fermentation and producing metabolites with protective effects against infectious and immune-related diseases [103]. Additionally, in a cross-sectional study conducted in public elementary schools in Brazil in children (7–11 years), it was found that several bacterial genera showed significant correlations with inflammatory cytokines, suggesting that UPF-induced microbiota changes may promote systemic inflammation. Dorea and *Subdoligranulum* were associated with IL-17A and IL-10; *Agathobacter* with IL-6, IL-10, and IFN-γ; and *Faecalibacterium* with IL-10, IFN-γ, and TNF-α [104].

##### Infant Formula

Although infant formulas are formulated to approximate the nutritional composition of human milk, notable differences have been described in the gut microbiota profiles of breastfed and formula-fed infants. While these products should not be conceptually equated with discretionary UPFs such as snack foods or sugar-sweetened beverages, their inclusion in this review is justified by their industrial processing and their potential influence on microbiota establishment during a critical developmental period. Importantly, they constitute a safe and nutritionally indispensable alternative when breastfeeding is not possible, contraindicated, or insufficient.

In this context, Forbes et al. [105] reported that formula feeding appears to promote microbiota profiles associated with overweight, characterized by greater microbial diversity and enrichment of *Bacteroidaceae*, whereas other complementary foods did not, instead being associated with lower diversity and enrichment of *Bifidobacteriaceae* and *Veillonellaceae* in healthy full-term infants.

In another study [87], it was suggested that diet was not the primary driver of gut microbiota maturation in healthy full-term infants. Interestingly, taxa commonly associated with human skin, such as *Propionibacterium*, *Staphylococcus*, *Gemella* and *Corynebacterium*, were found to be more dominant and persistent in infant microbiota, possibly reflecting frequent and close contact with maternal skin. However, these findings should be interpreted cautiously, given the very small size (n = 8, four by group). Ma et al. [106] observed that fecal microbial diversity was lower in breastfed infants than in formula-fed infants during early life but increased significantly after the introduction of solid foods. Notably, the lower microbial diversity associated with breastfeeding appeared to reflect a healthy gut pattern dominated by *Bifidobacteria*. The study also observed that different infant formulas exerted distinct effects on microbiota. A formula containing lower protein content, double the amount of docosahexaenoic acid/arachidonic acid and a postbiotic (thermally inactivated *Bifidobacterium animalis* subsp. Lactis) fostered a gut microbiota composition more similar to that of breastfed infants, in terms of richness and diversity of *Bacteroides*, *Bifidobacterium*, *Clostridium*, and *Lactobacillus* at 21 days, 2 and 6 months.

A similar relationship was observed in another randomized clinical trial, in which supplementation of infant formula with prebiotics appeared to promote gut maturation patterns more closely to those of breastfed infants [107]. In another randomized controlled trial involving healthy full-term infants, prebiotic-enriched formula increased both the abundance and relative proportion of *Bifidobacteria* [108]. Additionally, a prebiotic-supplemented formula was shown to mimic the effects of human milk in 362 healthy infants by promoting the growth of Bifidobacterium and Lactobacillus, and inhibiting the growth of Clostridium, contributing to a lower incidence of infant colic [109]. Another study evidenced that infant formula containing bovine milk-derived oligosaccharides combined with probiotics has a stronger bifidogenic effect than formula containing probiotics alone and produced microbial patterns more similar than that observed in breastfed healthy term infants [110].

The intestinal microecology of premature infants differs substantially from that of full-term infants. Among preterm infants, those who received breast milk showed a higher prevalence of *Clostridiales*, *Lactobacillales*, and *Bacillales*, and a lower prevalence of Enterobacteriaceae, compared with infants fed formula milk [111]. Another study reported that Firmicutes, Proteobacteria, and Actinobacteria accounted for more than 99% of the total bacteria in all preterm infants, with Bacteroides representing only 0.3% of the total microbial population [112]. The same study also found that breastfeeding increases the α-diversity of intestinal bacteria compared with formula feeding. Furthermore, Firmicutes abundance was higher in preterm infants fed formula than in those fed with breast milk, which may partially explain the greater weight gain commonly observed in premature infants during the first year of life [112].

However, it is also important to note that in certain populations, such as newborns experiencing weight loss, supplementation with early limited formula does not appear to interfere with the gut microbiota composition [113].

##### Other UPFs in Infant Nutrition

Numerous studies have shown that the transition from a milk-based diet to complementary foods alters the infant gut microbiota [114,115,116]. Increasing dietary diversity has a significant impact on microbiomes as solids are introduced [115]. Recent evidence suggests that the introduction of meat or iron-fortified cereals with fruit can enhance microbial biodiversity [116].

Plaza-Díaz et al. [114] investigated whether the consumption of infant cereals formulated with 50% whole grains and reduced sugar content could beneficially modulate the fecal microbiota of infants aged 4–7 months. Their results suggested that, compared with these reformulated products, cereals based on refined and hydrolyzed flours were associated with an unfavorable impact on gut microbiota composition. Specifically, intake of refined cereal-based products was linked to an increased abundance of anaerobic fermentative bacteria and a reduction in beneficial microbial balance, alongside a relative enrichment of potentially pathogenic *Escherichia* spp., suggesting a less desirable microbial profile during a critical window of gut microbiota development.

In a study by Faggiani et al. [90], involving 728 children from the MINA-Brazil birth cohort, researchers investigated whether UPF consumption negatively affects the diversity and abundance of the gut microbiota. The effects were more pronounced in children who had already been weaned. Regarding Bifidobacterium, a genus considered a key indicator of gut health in childhood [117], the findings remain inconclusive. However, another study showed a decline in Bifidobacterial abundance in children who consumed sweets, desserts, or infant formulas from one year of age onwards [118]. In this sense, the reductions in Bifidobacterium abundance during early life have been associated with an increased risk of obesity, T2D, metabolic disorders, and higher all-cause mortality in later stages of life, highlighting the potential long-term health implications of early microbial dysbiosis [119].

## 5. Biopsychosocial Pathway Model of UPF Impact

**Figure 2:**
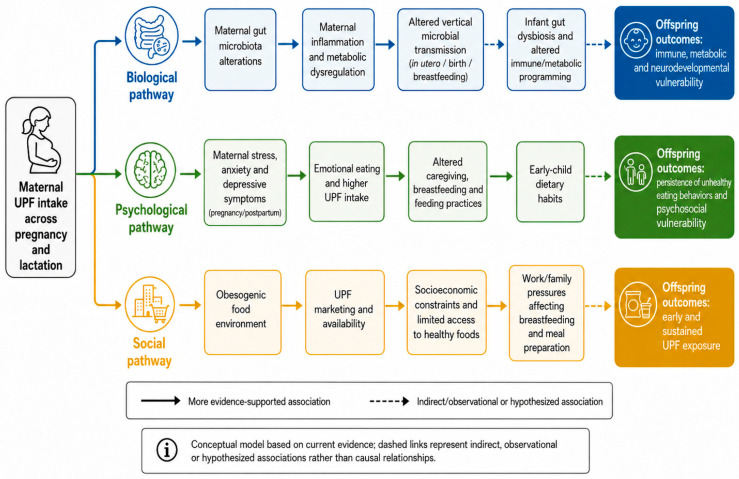
Biopsychosocial pathway model illustrating the impact of UPFs on maternal and infant health.

Given the interconnected biological, psychological, and social pathways through which UPF exposure influences maternal and child health, it is essential to examine not only what is affected, but how these factors interact and propagate risk. To better understand the consequences of UPF consumption during pregnancy and early childhood, we propose a biopsychosocial pathway model. This framework synthesizes the primary mechanisms through which UPFs influence maternal and child health across three key dimensions: biological, psychological, and social (Figure 2). Through this model, we illustrate how UPF consumption during pregnancy may initiate a cascade of interrelated biological, psychological, and social effects that ultimately shape the offspring health trajectory. Visualizing these pathways allows us to identify early intervention points to mitigate long-term risks.

On the biological axis, high UPF intake during pregnancy can disrupt maternal gut microbiota composition. Such alterations may impair vertical microbiota transmission during birth and breastfeeding, contributing to neonate dysbiosis. Early life disruptions have been associated with chronic inflammation, impaired immune modulation, and altered metabolic programming [120].

The psychological axis considers how maternal stress, anxiety, and depressive symptoms may increase emotional UPF consumption and, in turn, exacerbate dysbiosis and systemic inflammation. These psychological factors can also negatively influence caregiving practices and shape early dietary behaviors in children [121].

Finally, the social axis highlights the influence of obesogenic environments, aggressive food marketing, socioeconomic inequalities, and work-related barriers that limit breastfeeding and access to healthy, home-prepared meals. These social determinants create obstacles to optimal nutrition and perpetuate unhealthy feeding patterns.

Building upon this conceptual framework, we propose a transdisciplinary research model that identifies the key dimensions, biomarkers, and contextual factors that must be addressed to reduce the intergenerational burden of UPF-related disease.

## 6. Conclusions and Future Research Directions

Overall, this narrative review shows that the consumption of UPFs is a rapidly expanding global phenomenon, with a high and increasing contribution to energy intake in many countries, particularly in Western settings, and growing penetration in nutrition transition contexts. This increase is driven by structural changes in food systems, globalization, and the widespread availability of highly palatable, energy-dense, and nutrient-poor industrial products.

The synthesized findings highlight that UPF exposure is especially critical during sensitive stages of the life course, such as pregnancy and early childhood. In pregnant women, higher UPF intake is consistently associated with poorer overall diet quality, deficiencies in essential micronutrients, and increased risk of metabolic and obstetric complications, including excessive gestational weight gain, gestational diabetes, and preeclampsia. In addition, plausible biological mechanisms are emerging, including systemic inflammation and alterations in the gut microbiome, with potential consequences for fetal development.

In early childhood, the evidence points to a high and increasing contribution of UPFs to the diet, even among infants and young children, with relevant consequences for nutritional status, obesity risk, diet quality, and the early establishment of obesogenic dietary patterns. There is also evidence suggesting potential effects on neurodevelopment and immune–metabolic health, partly mediated by alterations in the gut microbiota during a critical window of biological maturation.

Across all stages, this review underscores the central role of the gut–microbiota–brain axis as an integrative mechanism underlying the effects of UPFs, together with psychological and social factors that modulate their consumption. In this regard, the proposed biopsychosocial model provides a relevant conceptual framework by situating UPF exposure within a complex system of biological, behavioral, and structural determinants.

In conclusion, the evidence synthesized suggests that high UPF consumption is not merely a marker of poor dietary quality, but a potential intergenerational risk factor for metabolic, immune, and neurodevelopmental health. Although most available studies are observational and do not allow causal inference, the consistency of associations and biological plausibility support the need for public health strategies aimed at reducing UPF exposure, particularly among women of reproductive age and in early childhood.

## Data Availability

No new data were created or analyzed in this study. Data sharing is not applicable to this article.

## Abbreviations

The following abbreviations are used in this manuscript:

UPF/UPFs	Ultra-Processed Food(s)
WHO	World Health Organization
BMI	Body Mass Index
CRP	C-reactive protein
PEI-UPF	Percentage of Energy Intake from Ultra-Processed Foods
IFN-γ	Interferon gamma
TNF-α	Tumor Necrosis Factor alpha
